# Poly(lactic acid)/Plasticizer/Nano-Silica Ternary Systems: Properties Evolution and Effects on Degradation Rate

**DOI:** 10.3390/nano13071284

**Published:** 2023-04-05

**Authors:** Roberta Capuano, Roberto Avolio, Rachele Castaldo, Mariacristina Cocca, Giovanni Dal Poggetto, Gennaro Gentile, Maria Emanuela Errico

**Affiliations:** 1 Institute for Polymers, Composites and Biomaterials—IPCB, National Research Council of Italy (CNR), Via Campi Flegrei 34, 80078 Pozzuoli, Italy; roberta.capuano@ipcb.cnr.it (R.C.); rachele.castaldo@ipcb.cnr.it (R.C.); mariacristina.cocca@ipcb.cnr.it (M.C.); giovanni.dalpoggetto@ipcb.cnr.it (G.D.P.); gennaro.gentile@ipcb.cnr.it (G.G.); 2 Department of Mechanical and Industrial Engineering—DIMI, University of Brescia, Via Branze 38, 25121 Brescia, Italy

**Keywords:** polymer-matrix nanocomposites, plasticization, silica nanoparticles, mechanical properties, biodegradable polymers, hydrolytic degradation

## Abstract

Plasticized nanocomposites based on poly(lactic acid) have been prepared by melt mixing following a two-step approach, adding two different oligomeric esters of lactic acid (OLAs) as plasticizers and fumed silica nanoparticles. The nanocomposites maintained a remarkable elongation at break in the presence of the nanoparticles, with no strong effects on modulus and strength. Measuring thermo-mechanical properties as a function of aging time revealed a progressive deterioration of properties, with the buildup of phase separation, related to the nature of the plasticizer. Materials containing hydroxyl-terminated OLA showed a higher stability of properties upon aging. On the contrary, a synergistic effect of the acid-terminated plasticizer and silica nanoparticles was pointed out, inducing an accelerated hydrolytic degradation of PLA: materials at high silica content exhibited a marked brittleness and a dramatic decrease of molecular weight after 16 weeks of aging.

## 1. Introduction

Poly(lactic acid) (PLA) is a biobased, biodegradable polyester showing good mechanical, thermal, and optical properties and a competitive cost, thus representing one of the most promising biopolymers for the substitution of oil-based plastic materials [1]. PLA is increasingly used in a wide range of applications, including biomedical materials [2], disposable products (kitchenware, packaging), and more durable products such as automotive parts and textiles [3,4,5]. The recent attention on the environmental risks caused by non-degradable plastics is boosting the market of biodegradable polymers, including PLA; however, some of its intrinsic characteristics represent an obstacle for a wider diffusion. In particular, PLA is inherently stiff and brittle and, for this reason, the realization of PLA-based materials with increased ductility and/or toughness is a widely investigated topic [6]. Among the different strategies developed to modify PLA mechanical response, including blending with other biopolymers, copolymerization, and the realization of composites, a convenient and effective way to reduce stiffness and increase ductility is through blending with plasticizers, that is, additives with a low temperature of glass transition and low stiffness that are able to modify the thermo-mechanical behavior of the host polymer matrix, increasing ductility [7]. Different additives have been successfully tested as plasticizers for PLA; however, to obtain materials with well-balanced properties, the chemical nature of the plasticizer, its concentration, and the processing conditions need to be carefully optimized. In fact, the improvement in ductility in most plasticized polymers often involves an undesired decrease in elastic modulus and strength. Moreover, a very good plasticizer/polymer miscibility is required to avoid a progressive phase separation over time, shown by most low molecular weight plasticizers. This separation process would result in unstable thermo-mechanical properties of the blend and in other undesired phenomena, such as a migration of the additive at the surface [8].

The use of organic or inorganic nanofillers has proven to be an effective way to tune the thermo-mechanical properties of polymeric materials, as demonstrated by a wide number of studies [9,10]. In particular, the interactions established among the surface of nanoparticles and the organic components can influence the behavior of polymers at a molecular level (chain mobility, conformation, relaxation), affecting macroscopic properties [11,12]. In polymer/plasticizer blends, the addition of proper nanofillers characterized by surface groups potentially interacting with the functional moieties of the polymer and/or the plasticizer can play a role in the phase segregation and/or migration processes, possibly leading to an increased stability of properties over time [13]. An assessment of the evolution of structure/morphology (buildup of phase separation) and thermo-mechanical properties (in particular ductility, strength, glass transition temperature) in this kind of ternary system, and a clarification of the mechanisms driving such evolution, would be of great importance, providing a tool to modulate the properties of plasticized PLA-based nanocomposites.

With this in mind, building on recent reports on PLA-based plasticized systems containing calcium carbonate nanoparticles [13], layered double hydroxides [14], and cellulose [15], we have investigated a new PLA-based ternary system containing oligomeric plasticizers and fumed silica nanoparticles. PLA nanocomposites containing silica nanoparticles of different natures have been widely investigated [16], mainly with the aim to obtain materials with enhanced thermo-mechanical response [17], thermal stability [18], and gas barrier properties [19]. Here, however, our aim was different: silica nanoparticles were selected to be introduced into PLA in combination with oligomeric plasticizers (OLAs) to investigate their effect on the properties of ternary systems and on their evolution in time. Few reports can be found in the literature that analyze PLA composites containing silica nanoparticles and plasticizers [20,21]; however, none of them provide an investigation of the time evolution of structure and properties.

Measuring the thermo-mechanical properties of PLA/OLA/silica ternary systems as a function of composition and aging over 16 weeks revealed an unexpectedly fast evolution of properties, strongly influenced by silica and related to PLA crystallization and OLAs phase separation. Molecular weight analysis highlighted a strong effect of both the additives and the nanoparticles on the hydrolytic degradation of PLA, suggesting a way to modulate not only properties but also the degradation rate of the polymer in ambient conditions.

## 2. Materials and Methods

Poly(lactic acid) (grade 4032D; <2 mol % of D stereoisomer) was provided by Nature Works.

Fumed silica nanoparticles (Aerosil 200, BET surface area 200 m^2^/g, coded as SiNP) were kindly supplied by Evonik Industries AG, Germany.

Esterified oligomers of L-lactic acid OLAs, hydroxyl terminated (OLA_OH), and carboxyl terminated (OA_COOH) were kindly supplied by Condensia Quìmica SA, Barcelona, Spain. Both esters have been characterized elsewhere [22].

Before use, PLA and OLAs were dried at 60°C in a vacuum oven for 24 h; SiNP were dried at 100 °C for 24 h.

### 2.1. Preparation of PLA Based Nanocomposites

PLA-based nanocomposites were realized by melt compounding, following a procedure in two steps:

- Silica nanoparticles (SiNP) were mixed with plasticizers (compositions are reported in Table 1) and were homogenized by stirring at 85 °C for 10 min, producing high silica content masterbatches;
- The masterbatches and PLA were then melt-mixed in a Plastograph EC (Brabender GmbH & Co.) batch mixer at 170 °C and 60 rpm for 10 min.

The amount of SiNP and OLA (see Table 1) in each masterbatch was predetermined as a function of the desired final concentration in the composites, keeping the PLA/OLA weight ratio at 80/20 and SiNP contents equal to 1, 3 or 5 wt%. The content of SiNP was selected based on our previous experience in nanocomposite preparation [23,24] and on literature data [16].

The ternary materials obtained by melt mixing were pelletized and molded at 175 °C, according to the following pressure program: 1 min of pre-melting with no pressure applied, 2 min of melting at minimum pressure (approximately 5 bar), and 2 min at 50 bar. Then, 2 mm thick sheets and ≈150 μm thick films were produced; all samples were rapidly cooled to room temperature by means of water circulating cooling plates (water temperature 20 °C, cooling time ≈ 3 min).

Neat PLA was subjected to the same process to be used as a reference.

### 2.2. Aging

PLA/OLAs/SiNP samples were aged in a Climacell 222 climatic chamber at 25 ± 0.1 °C and at a relative humidity of 50 ± 1%. The materials were characterized after 24 h (unaged samples) and after an aging of 2, 4, 8, 12 and 16 weeks.

### 2.3. Techniques

Transmission electron microscopy (TEM) was carried out using a FEI Tecnai G12 Spirit Twin (LaB_6_ source) equipped with an FEI Eagle 4k CCD camera (Thermo Fisher Scientific, Waltham, MA USA). Accelerating voltage was set at 120 kV. For the analysis, ultrathin sections (150 nm nominal thickness) of the different samples were obtained at room temperature by means of a Leica UC6 ultramicrotome.

Scanning electron microscopy (SEM) was carried out by means of an FEI Quanta 200 FEG microscope (Thermo Fisher Scientific, Waltham, MA, USA) in high vacuum mode, with an acceleration voltage ranging from 5 to 15 kV and using a secondary electron detector. Impact-fractured surfaces were prepared for SEM observation by coating them with an Au/Pd alloy using an Emitech K575X sputtering device.

Differential scanning calorimetric analysis (DSC) was performed using a TA-Q2000 differential scanning calorimeter (TA Instruments, New Castle, DE, USA) equipped with an RCS-90 cooling unit. The instrument was calibrated in terms of temperature and energy with pure indium. Approximately 5 mg of the samples was sealed into a Tzero^®^ aluminum pan and heated from −70 to 190 °C at 20 °C/min. High-purity nitrogen gas was fluxed at 20 mL/min during all measurements. Crystallinity was calculated according to Equation (1):

Xc = 100 (Hm − Hc)/Hm_0_, (1)

where Xc is the crystallinity index, Hm and Hc are the melting and crystallization enthalpies, respectively, normalized on the weight of the PLA content for each material. Hm_0_ is the melting enthalpy of completely crystalline PLA, 93.6 J/g [25].

Tensile tests were performed on dumb-bell specimens (0.6 mm^2^ cross section, 0.15 mm thickness, 28 mm gage length) at a cross-head speed of 10 mm/min by using a 5564 Instron testing machine (Instron Inc., Norwood, MA, USA), testing at least eight specimens per material. Test temperature was set to 27 ± 1 °C through an Instron 3119-005 environmental chamber. Mechanical parameters calculated included Young’s modulus (E), yield stress (σ_Y_), stress-at-break (σ_R_), and elongation-at-break (ε_R_).

Charpy impact tests were carried out on notched specimens by means of Ceast Resil Impactor instrumented pendulum (Instron Inc., Norwood, MA, USA) equipped with a DAS 4000 Acquisition System, using an impact energy of 3.6 J, an impact speed of 1 m/s, and a span length of 48.0 mm. Samples (8.0 mm wide, 2 mm thick, and 60 mm long) with a notch depth-to-width ratio of 0.3 were tested at a temperature of 27 ± 1 °C. Impact toughness and peak force were calculated as average values over at least four tested samples.

Gel Permeation Chromatography (GPC) analyses were performed in CHCl_3_ (Romil Ltd., Cambridge, UK) at 35 °C, with a flow rate of 0.8 mL/min and a runtime of 45 min, by means of a Malvern-Viscotek GPC MAX/TDA 305 triple detector array (Malvern Panalytical Ltd., Malvern, United Kingdom). The column set was composed of a precolumn and two Phenogel (Phenomenex, USA) columns, with exclusion limits of 10^6^ and 10^3^ Da, respectively. Selected samples with different aging times were dissolved in CHCl_3_ (about 5–6.5 mg/mL) and filtered through a 0.22 μm PTFE membrane filter before injection (100 µL). The chosen evaluation method was universal calibration, based on narrow polystyrene standards with a molecular weight ranging from 1.290 MDa to 1.700 kDa. All measurements were performed in duplicate.

Thermogravimetric analysis (TGA) was performed by means of a Perkin Elmer Pyris Diamond TG/DTA analyzer (Perkin Elmer Inc. Waltham, MA, USA) in nitrogen atmosphere (100 mL/min), using a linear ramp from 20 °C to 800 °C at 20 °C/min.

## 3. Results and Discussion

### 3.1. Masterbatches

Masterbatches, coded as OLA_OH_xS or OLA_COOH_xS (where x indicates the amount of SiNP), were prepared to promote potential physico-chemical interactions between hydroxyl (OLA_OH) or carboxyl (OLA_COOH) end groups of OLAs with hydroxyl surface moieties of SiNP. Then, to assess hypothesized interactions, the adsorption of the OLAs onto silica was evaluated through a selective extraction. A portion of the masterbatches with the highest nanoparticle content was suspended in warm acetone and centrifuged several times to separate and recover the insoluble phase, that is, the silica-rich fractions. TGA analysis was carried out on these fractions and, for comparison, on neat SiNP and neat OLAs, to estimate the amount of organic phase adsorbed onto SiNP. TGA curves and their derivative curves (DTG) are reported in the Supplementary Materials, Figure S1. Silica shows a slight weight loss as a function of temperature, without any sharp degradation step, as indicated by the absence of peaks in the DTG curve. This weight loss (about 0.5% at 600 °C) can be attributed to the condensation of surface hydroxyl groups of silica with the release of water. On the other hand, OLAs are completely degraded and volatilized in the same temperature range. The TGA curves of both insoluble fractions show clear weight loss steps, attributed to the degradation of adsorbed OLAs; degradation temperatures are higher than in neat OLAs as a consequence of adsorption onto silica.

Comparing the weight loss of neat SiNP with the weight loss of the insoluble fractions, the amount of adsorbed OLAs was determined as follows: about 2% in the case of OLA_OH and about 1% for OLA_COOH. This result differs from our previous study where CaCO_3_ particles, notwithstanding their low surface area, were able to adsorb a significant amount of OLAs. The low adsorption observed in these systems can be rationalized considering that the stability of organic layers adsorbed onto silica through hydrogen bonds is strongly dependent on molecular structure (functional groups, molecular weight) [12]. The H-bond driven adsorption of OLAs onto silica proved, then, to be less stable than the acid/based interactions that were at the basis of OLA adsorption onto calcium carbonate.

### 3.2. Unaged Materials

On the basis of our previous studies, a PLA/OLA ratio of 80/20 can be considered the optimal composition with which to assure adequate plasticization of PLA [22]. Therefore, in ternary systems, the PLA/OLA weight ratio was kept at 80/20, varying nanoparticles content between 1 and 5 wt%, as presented in Table 2. Materials were prepared by melt mixing and then films and sheets were obtained by compression molding.

**Figure 1:**
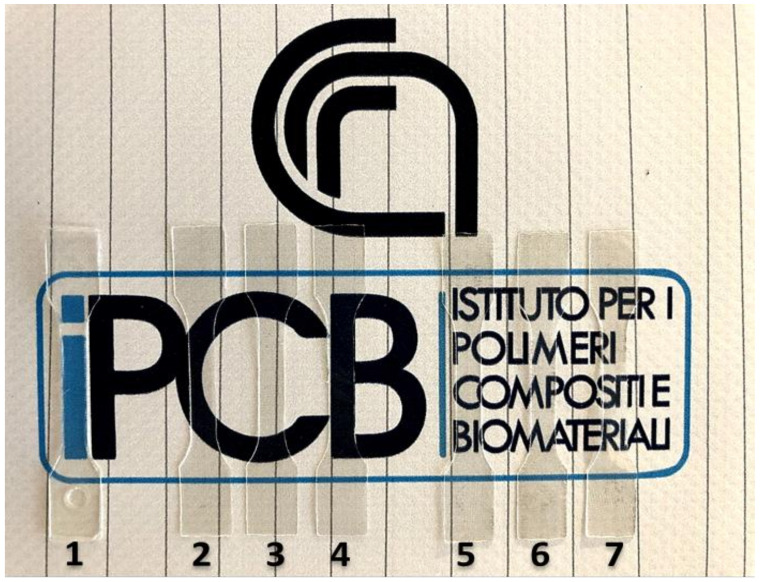
Picture of tensile specimens obtained from 150 µm films of neat PLA (1), S1_OH (2), S3_OH (3), S5_OH (4), S1_COOH (5), S3_COOH (6), S5_COOH (7).

Plasticized nanocomposite films are remarkably transparent even at the highest content of SiNP, as illustrated in Figure 1.

Compression molded materials were kept for 24 h at 25°C, 50% RH and were then subjected to morphological, thermal, and mechanical analyses.

**Figure 2:**
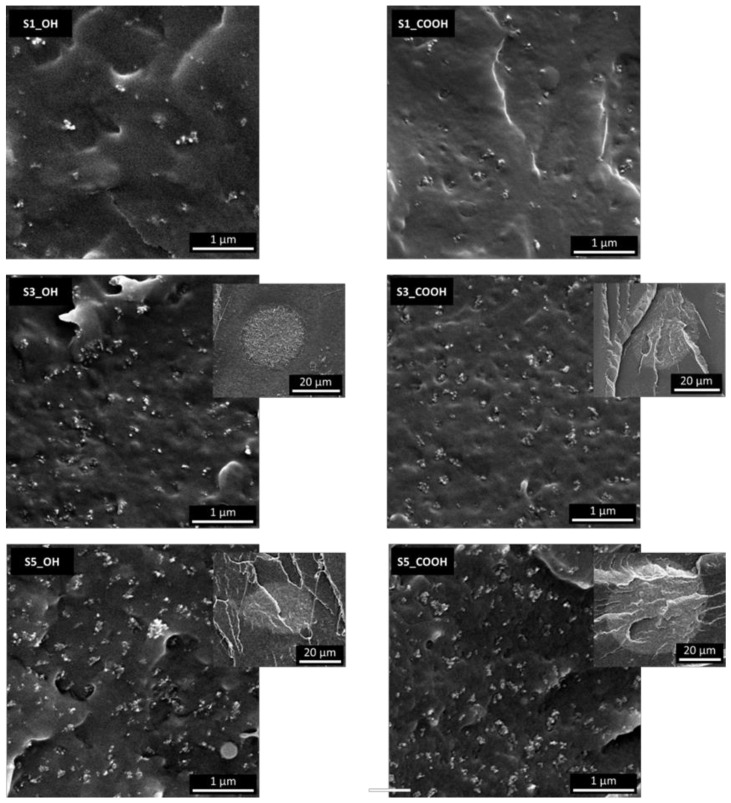
SEM micrographs of impact fracture surfaces of selected plasticized nanocomposites. Low magnification inserts are reported for samples 3S_X and 5S_X.

SEM micrographs were recorded on fractured surfaces to analyze the dispersion of SiNP in the polymeric phase and are reported in Figure 2.

The analysis of SEM micrographs reveals the presence of silica aggregates/agglomerates in the 50–200 nm range uniformly distributed in the polymeric phase, along with silica-rich globular domains with dimensions of tens of µm as evidenced in the S3 and S5 samples (the high silica content has been confirmed by EDX analyses reported in the Supplementary Materials, Figure S2). This morphology does not appear to be influenced by the nature of the plasticizer. This finding points out an imperfect mixing of SiNP into the polymer for silica content above 1 wt%, leading to a bimodal distribution of nanoparticles. The low melt viscosity of PLA/plasticizer blends, leading to a low shear force experienced by the filler during melt mixing, along with the low affinity of plasticizers for the surface of silica, are probably responsible for the imperfect dispersion observed [26]. It is worth noting that the nanometric aggregates formed by the well-dispersed fraction of SiNP have a size compatible with the fractal aggregates often observed in fumed silica nanocomposites (TEM analysis in Figure S3). Moreover, most aggregates are exposed at the surface of samples, indicating that the fracture propagated through the interface: this is a clear indication of a weak adhesion between the phases and in agreement with the observed clustering phenomena.

**Figure 3:**
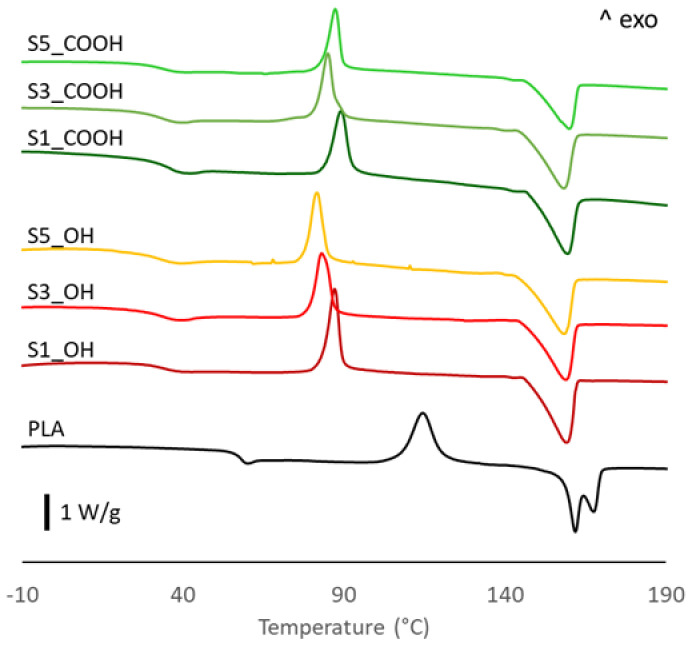
DSC traces of PLA and plasticized nanocomposites.

The thermal behavior of PLA-based systems was investigated by DSC analysis. The main thermal parameters are presented in Table 2 while the relative thermograms are illustrated in Figure 3. Before discussing the data, it is important to keep in mind that the calorimetric analysis was carried out on films obtained after rapid quenching from the melt and that the thermograms and evaluations refer to the first heating run, as detailed in Section 2. Thermograms of all plasticized nanocomposites show a single glass transition temperature whose value is significantly lower than that of neat PLA and that is only slightly affected by the nanoparticles content. As reported in our previous paper [22], this decrease can be ascribed to the plasticizing effect of OLA_OH and OLA_COOH oligomers intercalating between polymer chains. Moreover, the absence of any glass–rubber transition below 0 °C attributable to the OLA oligomers, whose Tg is expected in a range from −35 to −45 °C, suggests a good PLA/OLA miscibility. Observing the thermograms, an exothermic peak during the heating run appeared in all materials. This peak refers to the cold crystallization process, which is typical of PLA, mainly due to its slow-melt crystallization kinetic. It is interesting to note that the cold crystallization temperature is significantly lower and the transition peak is much narrower in all ternary systems with respect to PLA. This evidence highlights an acceleration of the cold crystallization process, which could be attributed to the effects of both OLAs and SiNP. On the one hand, in fact, plasticizers increase PLA chain mobility, favoring the rearrangements required for crystal formation [7], a phenomenon that was clearly observed in binary PLA/OLA systems [22]. On the other hand, the decrease of cold crystallization temperature at higher silica content suggests a nucleation effect of SiNP [27,28,29].

Concerning the melting behavior, neat PLA exhibits a peculiar double melting peak, with two well separated maxima centered at 162 and 168 °C. In the literature, two theories have been proposed to explain the double melting peak sometimes observed in PLA samples: the “lamellar thickness” model and the “melting-recrystallization” model [30]. The first model hypothesizes that the two melting endotherms are related to the presence of two families of crystal lamellae differing in thickness, with thinner lamellae showing a lower melting temperature than thicker ones. The second model relates the low temperature endotherm to the melting of disordered crystals initially present in the sample, immediately followed by recrystallization in more ordered crystalline structures that are then melted at a higher temperature. The disordered crystalline phase of PLA, denoted as α′, is a metastable form produced by crystallization from the melt in specific conditions [31,32]. While a detailed investigation of the different crystalline structures of PLA is beyond the scope of this work, we note that all plasticized nanocomposites showed a single, broad melting peak, centered at a temperature very close to the lower melting peak of neat PLA. This finding is in line with our previous study on binary PLA-OLA systems [22], so it can be mainly attributed to the action of plasticizers, which appears to favor the formation of the low-melting crystalline form of PLA. This finding can be related to the high concentration of OLAs which, while increasing the segmental mobility of polymer chains and, thus, the crystallization rate, accumulate at the front of growing spherulites, representing an obstacle to long-range ordering and, then, favoring more defective crystalline structures [33].

Finally, the crystallinity of pristine films, calculated according to Equation (1), shows a relevant increase in all ternary systems, with values up to four times that of neat PLA. Such a large increase in crystallinity content was not observed in binary PLA/OLA systems [22]. These findings, then, further support the role of SiNP in promoting the crystallization process of PLA, in synergy with the already-discussed effect of plasticizers.

Mechanical properties of PLA and ternary systems were measured at a low deformation rate in tensile mode; results are reported in Table 3. OLAs at 20 wt% were effective in plasticizing the nanocomposites, as all PLA-based materials show a relevant increase of the ultimate elongation, up to a value of 240%, and at the same time a decrease in stiffness. The elongation values recorded are remarkable for composites containing significant amounts of inorganic nanoparticles. The presence of silica did not show a clear reinforcing effect on the composites: on the contrary, modulus and strength decreased in both _OH and _COOH systems with increasing silica content. Materials containing the _OH plasticizer seem to be more affected by this “overplasticization” effect, showing lower modulus and strength at all silica contents. These findings suggest an effect of SiNP on plasticizer distribution in the nanocomposites that can be rationalized considering the strong increase in crystallinity recorded by thermal analyses. Silica particles can show a nucleating effect on PLA, as pointed out in previous reports [27,28], resulting in an increased crystallization rate and higher overall crystallinity. As crystalline domains of PLA cannot accommodate OLA molecules, crystallization will correspond to an increased concentration of plasticizer in the remaining amorphous phase, leading to lower glass transition temperature and lower stiffness—the “overplasticization” effect observed. In this respect, the weak interactions observed between OLAs and silica play an important role. In our previous paper [13], where nanoparticles showing strong interactions with OLAs were used, we evidenced an increased concentration of the plasticizer onto particle surfaces and only minor changes in the crystallization behavior were observed in such systems, in spite of the nucleating effect reported for calcium carbonate [34,35]. In the present system, OLAs do not strongly adsorb onto silica, leaving nanoparticle surfaces exposed to PLA, which are then able to promote crystallization efficiently.

The hypothesized uneven distribution of the plasticizer has implications for the evolution of properties with time, as further discussed in the next section, dealing with the effects of aging.

### 3.3. Aging Effects on Properties

Aging of PLA and ternary systems was carried out in a climatic chamber at 25 °C and 50% RH, reproducing storage in standardized ambient conditions. Thermo-mechanical properties were tested after 2, 4, 8, 12, and 16 weeks of an aging time (ta) to gather information on the effect of the SiNP particles on the evolution of properties.

**Figure 4:**
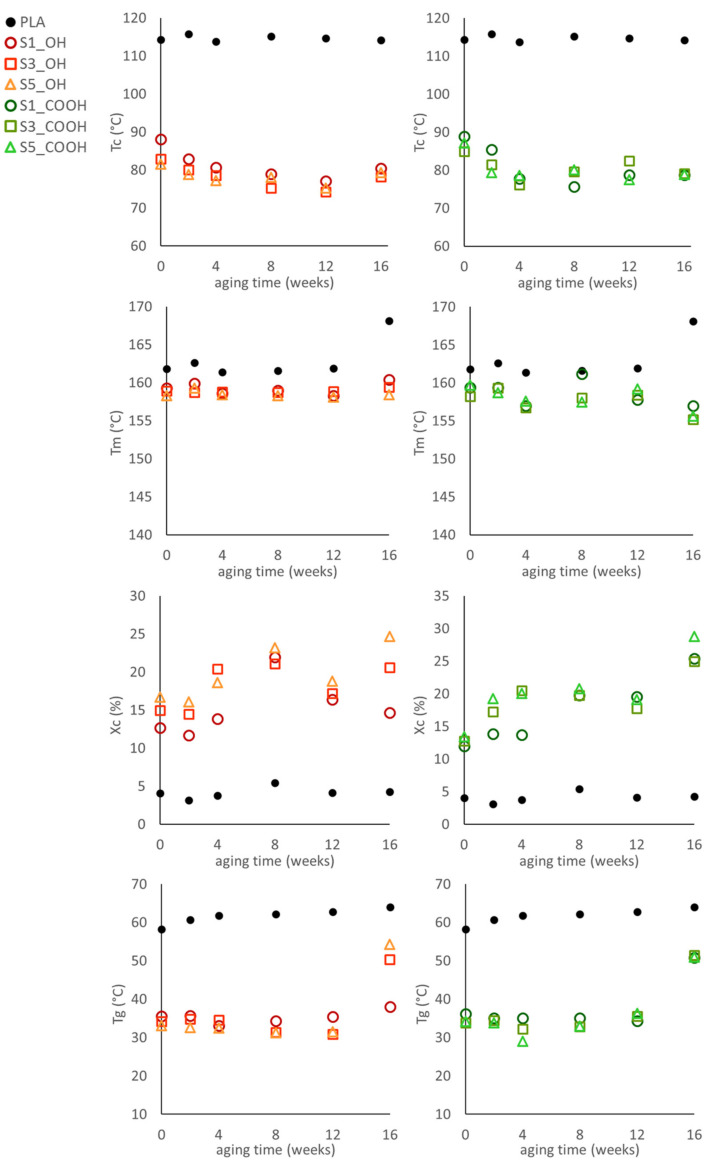
Calorimetric parameters: cold crystallization temperature (T_cc_), melting temperature (T_m_), crystallinity (X_c_), and glass transition temperature (T_g_) of the plasticized nanocomposites as a function of aging time. Estimated errors are ±1 °C for temperatures and ±2% for crystallinity content.

The main DSC parameters, recorded as a function of aging time t_a_, are reported in Figure 4.

DSC analysis of aged materials showed a substantial stability of melting temperature and glass transition temperature up to 12 weeks of aging. On the contrary, the cold crystallization temperature slightly decreases while the crystallinity content showed a general increase for all systems over the same aging period. At the longest aging time, however, stronger variations were recorded in thermal parameters: the glass transition of most materials increased to values close to the T_g_ of neat PLA, especially at higher silica content and in systems containing OLA_COOH as a plasticizer. The crystallinity also showed strong variations among samples aged for 16 weeks; _COOH-based materials were more affected.

These findings point out a drastic evolution in thermal properties over time, suggesting a phase separation of the PLA/OLA systems after 16 weeks. This process is apparently accelerated by the presence of SiNP.

**Figure 5:**
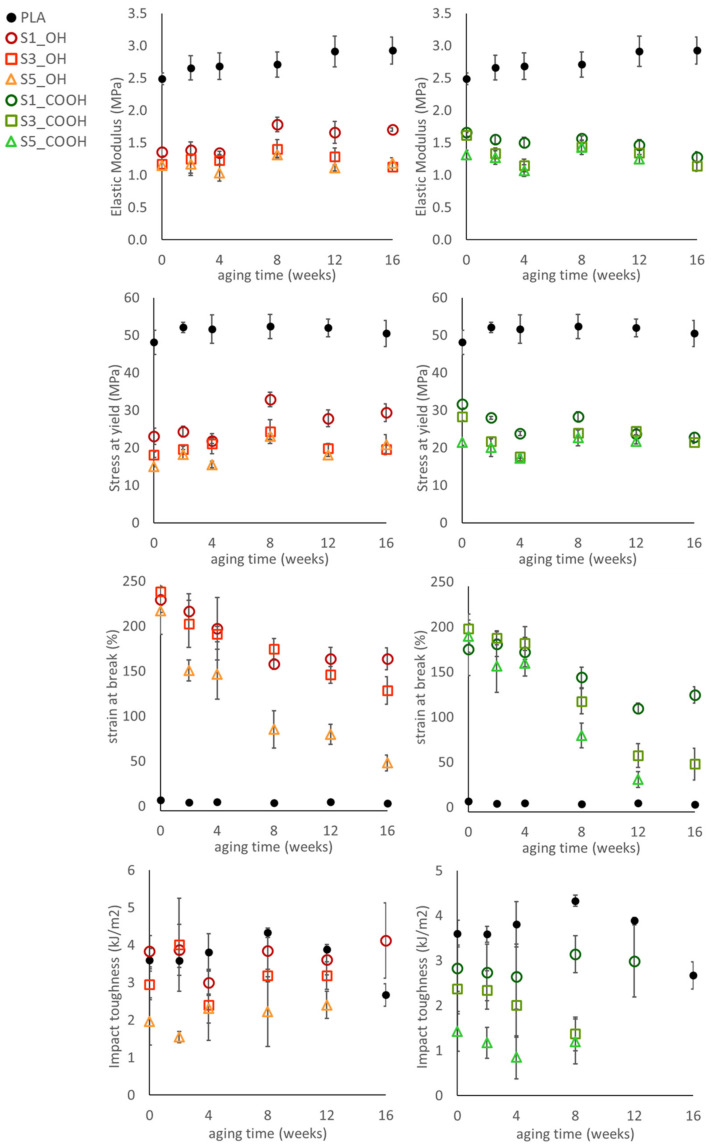
Evolution of the tensile and impact properties of the plasticized nanocomposites during aging.

The evidence of a phase evolution with aging time is also reflected by the trend of mechanical parameters of ternary systems, reported in Figure 5.

Analyzing the elastic modulus and the yield stress of plasticized nanocomposites, the general behavior observed in unaged samples was confirmed. Both parameters showed, indeed, a negative correlation with silica content at all aging times, irrespective of the plasticizer used. Upon aging, modulus and yield stress showed some fluctuation; in particular, a general stiffening was recorded on all samples, with different intensities, after 8 weeks of aging. However, no clear trend was observed up to the longer aging time.

The consequences of phase evolution are better illustrated by analyzing the trend of elongation-at-break, as this parameter is very sensitive to the presence of defects/heterogeneities into the materials. Elongation, indeed, shows large variations over time: a general decrease has been recorded for aging time up to 8 weeks; then, two subsets of composites with different behavior can be identified: S1_OH, S3_OH and S1_COOH showed a stabilization of elongation up to the longest aging time; S3_COOH and materials with 5% silica, on the contrary, showed an increasingly fragile behavior to the point that the sample S5_COOH at 16 weeks was so brittle it could not be tested.

The presence of SiNP at high concentrations, in principle, can be responsible for a premature failure; particle clusters, evidenced by morphological analysis, can in fact act as defects triggering the fracture. However, this mechanism alone does not fully explain the fast decrease of elongation and the embrittlement recorded in samples with high silica content, which is evidently an indication of structural/morphological changes developing in plasticized nanocomposites.

Charpy impact toughness of the plasticized nanocomposites gives further insights on the effect of silica and the strong changes occurring in the materials upon aging. Samples with higher silica content showed the lowest impact toughness: this finding corroborates the effect of silica on plasticizer distribution, as suggested by thermal analysis and discussed in Section 3.2. Low molecular weight plasticizers at high concentration are, in fact, often detrimental to impact resistance [36]. The evolution of impact toughness with aging time correlates with the proposed hypothesis of the structural evolution of materials: while toughness values show only slight fluctuations over time, within experimental error, from 12 weeks of aging some materials (S3_COOH and S5_COOH) begin to become too brittle to be tested (the load level during fracture was below the automatic trigger limit of the testing machine employed). Furthermore, at 16 weeks, only the S1_OH sample provided meaningful results in the Charpy impact test.

**Figure 6:**
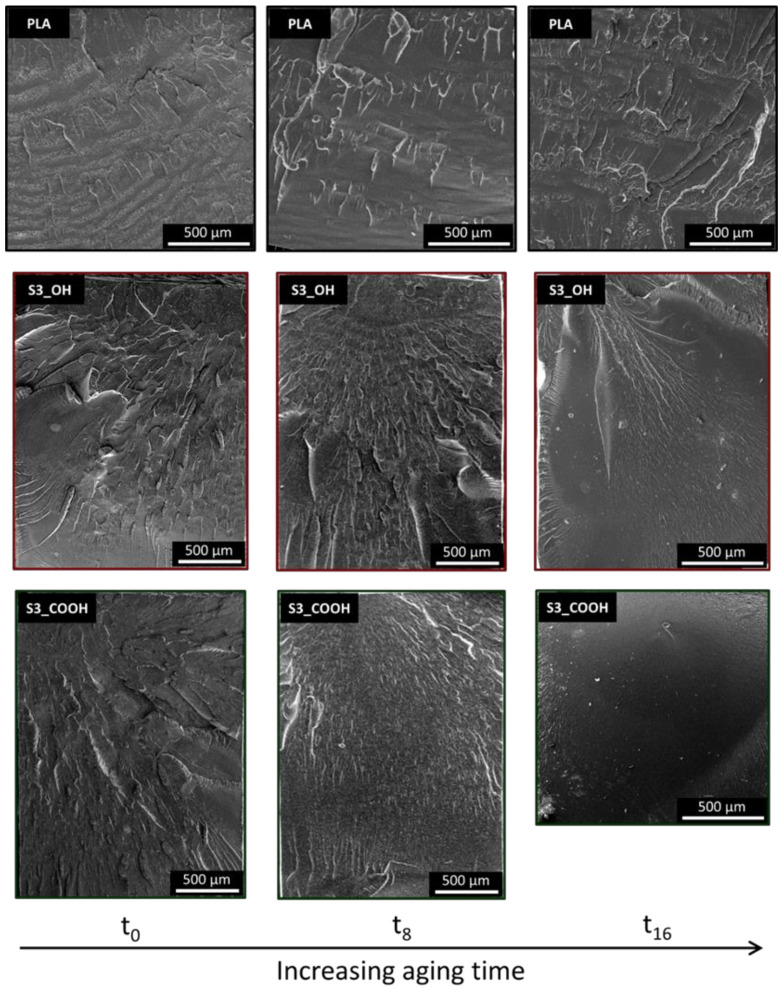
SEM micrographs of representative samples at different aging times. Fracture propagation direction is from the upper to the lower side of each micrograph.

To provide more information on the fracture behavior observed, fracture surfaces were analyzed by SEM, as reported in Figure 6. Neat PLA samples showed only minor changes in their fracture surfaces upon aging, with the presence of zones with increased roughness (symptomatic of a localized plastic deformation) mainly organized perpendicularly to the fracture propagation direction. In the plasticized nanocomposites up to 8 weeks of aging, the main features observed are fracture lines propagating along (parallel to) the direction of fracture, or radially from the fracture starting point. The surfaces are more irregular, probably due to the presence of silica nanoparticles and clusters, but the edges are sharp with little evidence of plastic deformation. At the longest aging time, the fracture surfaces of the nanocomposites shift towards a smooth, featureless appearance, showing a fast propagation of the fracture front with negligible deformation of the PLA, that is, an extremely brittle behavior. These observations are in line with the results of the Charpy impact tests, which demonstrated a lower toughness in most nanocomposites and a progressive embrittlement over time.

Overall, the main results obtained on aged materials can be summarized as:

- Thermal analysis suggests the build-up of phase separation, becoming evident for most samples at 16 weeks of aging;
- The stiffness, strength, and elongation of plasticized nanocomposites are influenced by silica in a non-obvious way, with higher silica content resulting in lower stiffness at all aging times;
- The ultimate elongation declines for all samples, and this decline is enhanced by the OLA_COOH plasticizer and by a higher silica content;
- Impact toughness follows a similar trend, with an evolution towards a brittle behavior that is faster for the _COOH series and for high silica content.

All data collected point out a complex interplay between plasticizer nature, silica content, and aging time, driving the morphological evolution of the ternary systems. Due to the strong evidence of the embrittlement of materials, the extent of which seems too large to be ascribed only to the progressive clustering and phase separation of the plasticizer, the molecular weight (MW) of the PLA phase was analyzed by means of GPC tests on samples at different aging times. Neat PLA and pristine, unaged samples were not analyzed as we demonstrated a negligible effect of the plasticizers on molecular weight during melt processing [22]. The results of GPC analyses on plasticized nanocomposites are reported in Table 4.

A decrease of MW has been recorded in all samples, strongly correlated to the presence of silica—that means that MW recorded in plasticized nanocomposites is lower at higher SiNP content. The nature of the plasticizer also shows a strong effect on molecular weight, as samples containing OLA_COOH always show a much lower MW.

The different effect induced by the two plasticizers can be rationalized considering that OLA_COOH has a free carboxylic acid group while the residual acidity of OLA_OH is very low [22]. This acidic character can catalyze the hydrolysis/transesterification of PLA [37]. The silica surface also has a slightly acidic behavior [38] and could, in principle, provide a further contribution to PLA degradation. However, the main factor accelerating the hydrolysis in samples with higher SiNP content can be ascribed to the increased crystallinity recorded at higher SiNP content. This increase, as discussed, entails a higher concentration of plasticizers in the amorphous regions, resulting in a higher hydrolysis rate induced by OLAs. The trend of molecular weight recorded reveals a strict relation with the thermal and mechanical behavior observed, especially at the longest aging time, being responsible for the embrittlement and for the phase separation evidenced.

In previous reports, where the same plasticizers have been used in combination with calcium carbonate nanoparticles, no strong evidence of embrittlement was observed even for longer aging times [13]. Then, it can be assessed that the evolution of properties observed in the present systems should be ascribed to the negligible SiNP/plasticizers interactions, leading to a de-stabilization of structure (accumulation of plasticizers due to higher crystallinity, development of phase separation, and strong reduction of PLA molecular weight due to the promotion of hydrolysis) in silica-containing systems. Then, these findings underline that, with an appropriate selection of organic additives (plasticizers or small molecules with the desired functional groups) and nanoparticles with specific surface properties, not only can the thermo-mechanical properties of PLA-based materials be modulated, but also the hydrolytic degradation of such systems can be tuned.

The control of degradation and, of course, bio-degradation rate is a key point to boost the penetration of PLA-based materials in new industrial fields. As an example, the acceleration of PLA degradation is of particular relevance in the light of recent reports, highlighting the very slow degradation of PLA in environmental conditions [39]. The results obtained in this work could then provide a tool for the design of PLA-based ternary materials with a careful control of both the thermo-mechanical properties and their stability in time, while at the same time tuning the rate of hydrolysis to ensure an easier degradation of products. Further investigations will be carried out to assess the influence of ambient conditions (composting, soil, water exposure) on degradation rate.

## 4. Conclusions

In this work, the realization of PLA-based plasticized nanocomposites, containing two different oligomeric esters of lactic acid as plasticizers and fumed silica nanoparticles, has been investigated. In freshly prepared materials, the nanoparticles showed a nucleation effect on PLA, in combination with the action of plasticizers. The increased crystallinity was, however, correlated to an “overplasticization” due to the accumulation of plasticizer molecules in the amorphous matrix surrounding crystals. As a consequence, the presence of silica did not lead to a mechanical reinforcement of the nanocomposites. Thermo-mechanical properties were investigated as a function of aging, carried out in controlled temperature and humidity conditions for up to 16 weeks. The materials revealed strong fluctuations in both thermal and mechanical parameters and a deterioration of mechanical response over time, suggesting the development of a phase separation of the plasticizers. A strong decrease of PLA molecular weight as a function of aging time was also observed, in particular in the presence of OLA-COOH, responsible for the faster degradation of properties recorded in this system. These findings were rationalized and attributed to the low affinity of OLAs towards silica surface, leading to the discussed nucleation and overplasticization effects, driving the accumulation and then phase separation of OLAs. In these conditions, the catalytic effect of carboxylic groups of OLA_COOH for the hydrolysis of PLA has been maximized in the areas of accumulation of the plasticizer, leading to an accelerated degradation of the polymer. The analysis of the mechanisms of interplay among nanoparticles, plasticizers, and PLA and their consequences for molecular structure and properties allowed us to gain a better understanding on the behavior of ternary systems, also in comparison with the results of previous reports [13]. Such mechanisms can thus be exploited to design materials with defined properties, in particular with a tunable degradation rate, which is a key property for many applications of PLA-based materials.

## Figures and Tables

**Table 1:** Composition of OLA/silica masterbatches.

Masterbatch	OLA_OH (wt%)	OLA_COOH (wt%)	SiNP (wt%)
OLA_OH_1S	95.2		4.8
OLA_OH_3S	86.6		13.4
OLA_OH_5S	79.2		20.8
OLA_COOH_1S		95.2	4.8
OLA_COOH_3S		86.6	13.4
OLA_COOH_3S		79.2	20.8

**Table 2:** Composition of nanocomposites and calorimetric (DSC) parameters: glass transition temperature (T_g_), cold crystallization temperature (T_c_), melting temperature (T_m_), and crystallinity content (X_c_). Estimated errors are ±1 °C for temperatures and ±2% for crystallinity content.

	SiNP (wt%)	OLA_OH (wt%)	OLA_COOH (wt%)	T_g_ (°C)	T_c_ (°C)	T_m1_-T_m2_ (°C)	X_c_ (%)
PLA	-	-	-	58	114	162–168	4
S1_OH	1	20	-	36	88	159	13
S3_OH	3	20	-	34	83	159	15
S5_OH	5	20	-	33	82	159	17
S1_COOH	1	-	20	36	89	159	12
S3_COOH	3	-	20	34	85	158	13
S5_COOH	5	-	20	34	87	160	13

**Table 3:** Stress-at-yield (σ_y_), elastic modulus (E), and strain-at-break (ε_R_) calculated from tensile test.

	σ_y_ (MPa)	E (MPa)	ε_R_ (%)
PLA	48 ± 3	2490 ± 90	6 ± 2
S1_OH	23 ± 2	1360 ± 60	230 ± 20
S3_OH	18 ± 1	1170 ± 60	240 ±20
S5_OH	15 ± 1	1150 ± 60	220 ± 30
S1_COOH	32 ± 1	1660 ± 60	150 ± 10
S3_COOH	28 ± 1	1620 ± 40	200 ± 20
S5_COOH	21 ± 1	1320 ± 60	190 ± 20

**Table 4:** GPC number-averaged (M_n_) and weight-averaged (M_w_) molecular weight of the PLA phase for samples at different aging times.

t_a_		Molecular Weight (kDa)					
		S1_OH	S3_OH	S5_OH	S1_COOH	S3_COOH	S5_COOH
8	M_w_ M_n_	62.9 20.9	44.9 19.2	32.7 17.3	38.2 17.2	31.5 16.5	26.1 13.7
16	M_w_ M_n_	48.6 15.8	45.6 18.2	25.1 13.1	37.3 15.9	14.0 5.9	10.6 5.3

## Data Availability

Not applicable.

## Supplementary Materials

The following supporting information can be downloaded at: https://www.mdpi.com/article/10.3390/nano13071284/s1, Figure S1: TGA and their derivative (DTG) curves of neat SiNP, neat OLA_OH and OLA_COOH, compared to the curves recorded on the insoluble fraction recovered from OLA_SiNP masterbatches after solvent extraction.; Figure S2: SEM micrograph of the fracture surface of a representative sample, with EDX elemental map showing the distribution of carbon and silicon atoms; Figure S3: TEM micrographs at different magnification of samples S3_OH (a, b, c) and S3_COOH (d, e, f).
