# Supplementary material for: Poly(lactic acid)/Plasticizer/Nano-Silica Ternary Systems: Properties Evolution and Effects on Degradation Rate
Source: Nanomaterials (Basel). 2023 Apr 5;13(7):1284. doi: 10.3390/nano13071284 (PMC10097254; doi:10.3390/nano13071284)
Supplement: Supplementary file 1 [file nanomaterials-13-01284-s001.zip › nanomaterials-2267053-supplementary.pdf]

SUPPLEMENTARY MATERIALS

Poly(lactic acid)/plasticizer/nano-silica ternary systems: properties evolution and effects on degradation rate

Roberta Capuano [1,2], Roberto Avolio [1,*], Rachele Castaldo [1], Mariacristina Cocca [1], Giovanni Dal Poggetto [1], Gennaro Gentile [1], Maria Emanuela Errico [1,*]

[1] Institute for Polymers, Composites and Biomaterials—IPCB, National Research Council of Italy (CNR), Via Campi Flegrei 34, 80078 Pozzuoli, Italy; roberta.capuano@ipcb.cnr.it (R.C.); rachele.castaldo@ipcb.cnr.it (R.C.); mariacristina.cocca@ipcb.cnr.it (M.C.); giovanni.dalpoggetto@ipcb.cnr.it (G.D.P.); gennaro.gentile@ipcb.cnr.it (G.G.)

[2] Department of Mechanical and Industrial Engineering—DIMI, University of Brescia, Via Branze 38, 25121 Brescia, Italy

* Correspondence: roberto.avolio@ipcb.cnr.it (R.A.); mariaemanuela.errico@ipcb.cnr.it (M.E.E.)

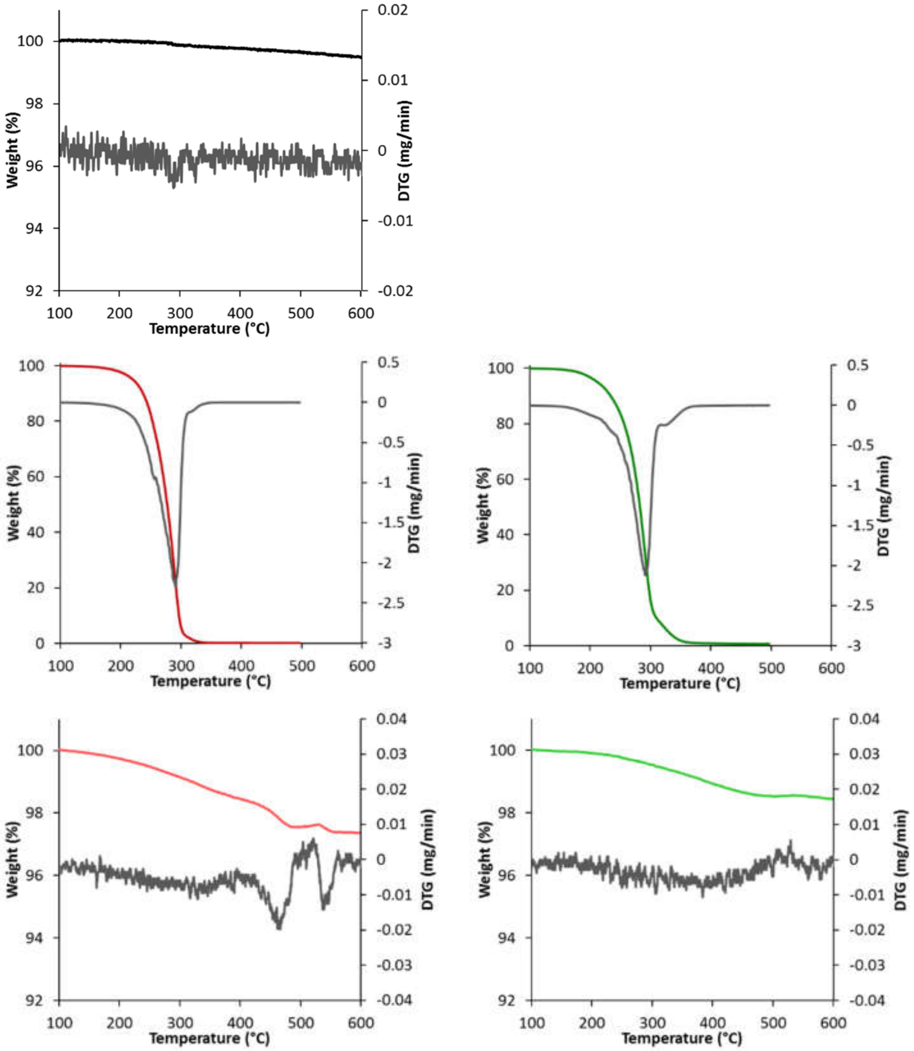

Figure S1. TGA and their derivative (DTG) curves of neat SiNP, neat OLA_OH and OLA_COOH, compared to the curves recorded on the insoluble fraction recovered from OLA_SiNP masterbatches after solvent extraction.

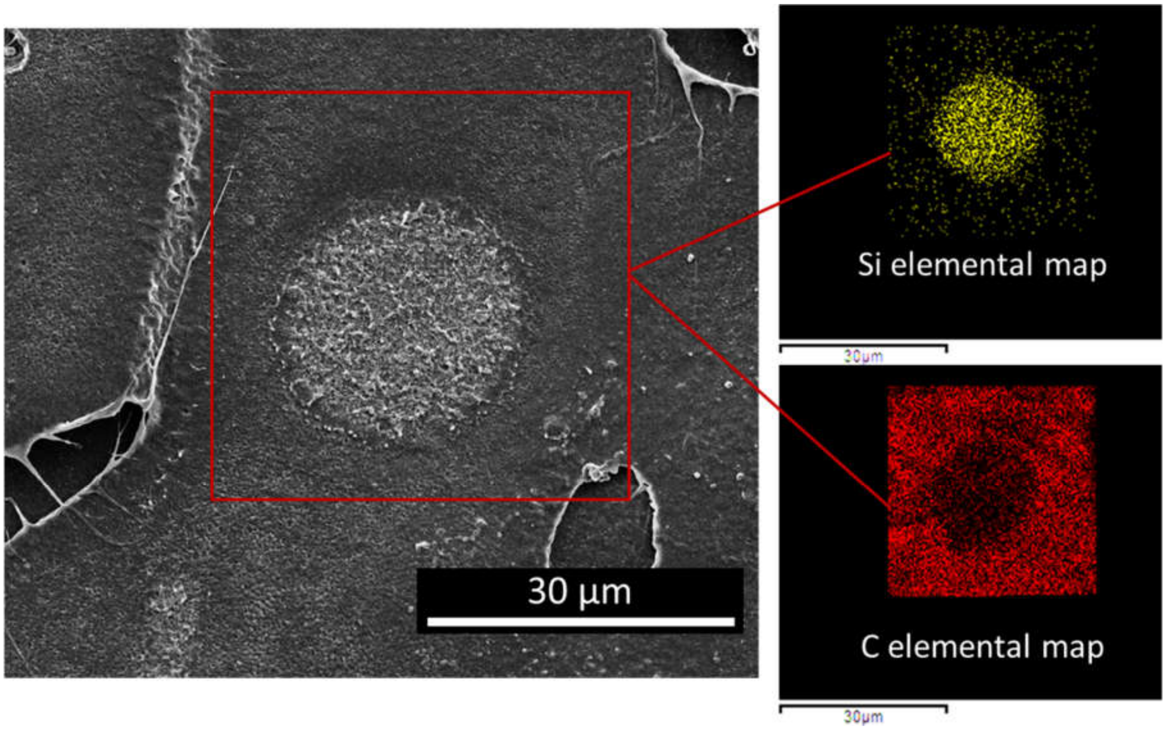

Figure S2. SEM micrograph of the fracture surface of a representative sample, with EDX elemental map showing the distribution of carbon and silicon atoms.

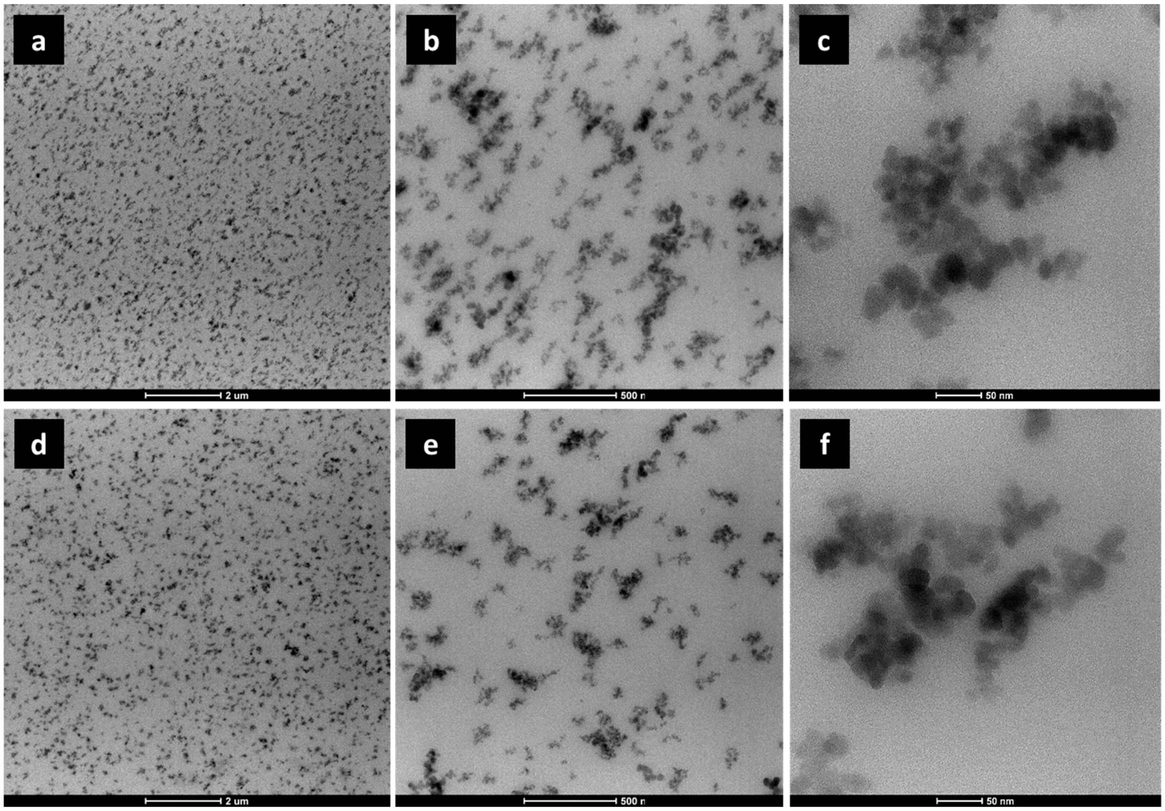

Figure S3. TEM micrographs at different magnification of samples S3_OH (a, b, c) and S3_COOH (d, e, f)
